# Towards the Design of Efficient and Secure Architecture for Software-Defined Vehicular Networks

**DOI:** 10.3390/s21113902

**Published:** 2021-06-05

**Authors:** Muhammad Adnan, Jawaid Iqbal, Abdul Waheed, Noor Ul Amin, Mahdi Zareei, Asif Umer, Ehab Mahmoud Mohamed

**Affiliations:** 1 Department of Information Technology, Hazara University Mansehra, Mansehra 21120, Pakistan; adnan25408@gmail.com (M.A.); jawaid5825@gmail.com (J.I.); namin@hu.edu.pk (N.U.A.); asifumer@hu.edu.pk (A.U.); 2 Department of Computer Science, Capital University of Science and Technology, Islamabad 44000, Pakistan; 3 School of Electrical and Computer Engineering, Seoul National University, Seoul 08826, Korea; 4 Tecnologico de Monterrey, School of Engineering and Sciences, Zapopan 45201, Mexico; m.zareei@tec.mx; 5 Electrical Engineering Department, College of Engineering, Prince Sattam Bin Abdulaziz University, Wadi Addwasir 11991, Saudi Arabia; ehab_mahmoud@aswu.edu.eg; 6 Electrical Engineering Department, Faculty of Engineering, Aswan University, Aswan 81542, Egypt

**Keywords:** VANETs, SDVN, security, public key infrastructure (PKI), digital signature, AVISPA

## Abstract

Recently, by the rapid development of Vehicular Ad Hoc Networks (VANETs) and the advancement of Software Defined Networking (SDN) as an emerging technology, the Software-Defined Vehicular Network (SDVN) has a tremendous attraction in the academia and research community. SDN’s unique properties and features, such as its flexibility, programmability, and centralized control, make the network scalable and straightforward. In VANETs, traffic management and secure communication of vehicle information using the public network are the main research dimensions in the current era for the researchers to be considered while designing an efficient and secure VANETs architecture. This paper highlights the possible identified threat vectors and efficiently resolves the network vulnerabilities to design a novel and secure hierarchic architecture for SDVN. To solve the above problem, we proposed a Public Key Infrastructure-based digital signature model for efficient and secure communication from Vehicle to Vehicle. We also used the public key authority infrastructure for Vehicle to Infrastructure and the three-way handshake method for secure session creation and secure data communication in the SDN controller. The proposed security is validated through the well-known simulation tool AVISPA. Additionally, a formal security model is applied to validate the design hierarchic architecture’s fundamental security properties for SDVN in an efficient and desirable way. In a comparative analysis, we prove that our proposed scheme fulfills all the essential security properties compared to other states of the art schemes.

## 1. Introduction

**Figure 1:**
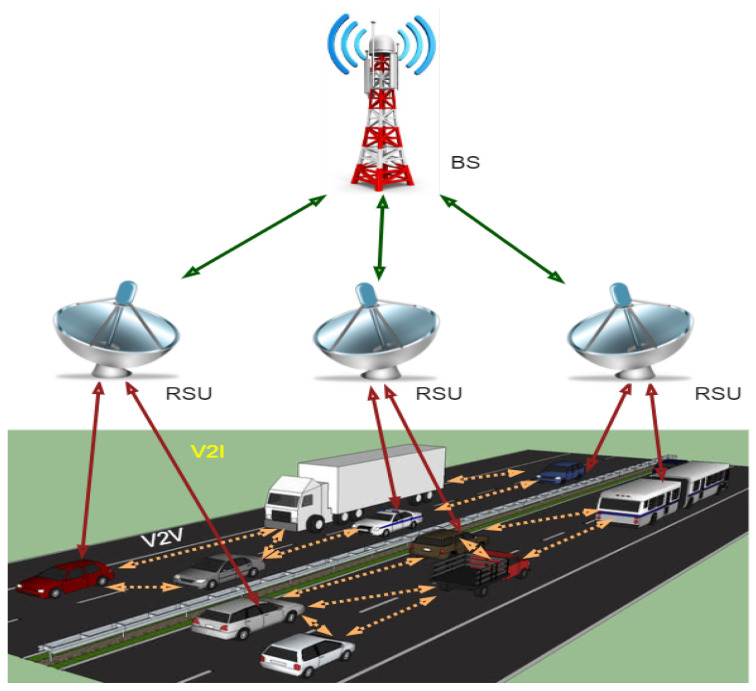
Components of VANET and its communications.

Due to rapid development in information technology and the increased demand for intelligent transportation systems, vehicular networks attract researchers’ interest in the more considerable interest of users. ITS is related to transportation infrastructures and advanced vehicles for a specific purpose such as driving safer, efficiently, and securely using information and communications technology [1]. VANET is an integral element of ITS and a particular Mobile Ad Hoc Network (MANET). There are three communication types of VANETs: Vehicle to Vehicle (V2V), Vehicle to Road (V2R), and Vehicle to Infrastructure (V2I). There are several communication technologies through which VANET can communicate, such as Dedicated Short-Range Communication (DSRC), which provides the opportunity for communication among ITS components (vehicles, infrastructure), and WAVE (Wireless Access in Vehicular Environment) that defines MAC/PHY protocols and standards used for vehicular communication [1]. The researchers are trying to design new VANETs architectures and develop protocols for routing and security, applications, and simulation tools to efficiently and securely improve communication and control traffic management. Currently, VANETs have faced many difficulties, such as less scalability, less flexibility, and less programmability for implementing the services in a large-scale environment. The management of the network and traffic in VANETs is challenging due to the lack of inefficient routing and dynamic behavior of VANETs, the network is congested, and the problem of network throughput is created [2]. Therefore, a new networking strategy was created, known as SDN. It is an emerging technology to control and manage the network in a programmable way to separate the control plane from the data plane to improve the networks’ overall performance. In addition, the SDN’s basic concept separates control logic from hardware switches. Hence, SDN’s whole idea is to take care of limitations by separating the hardware’s application and operating system for efficient utilization of system resources and enhancing the lifetime of the networks. To flow traffic within the entire network, SDN separates data plan from control plan using centralized network controllers [3]. SDN technology provides organizations with an opportunity to build innovative APIs and offer new services and business representation. SDN’s unique features are its flexibility, programmability, and centralized control, making the network’s design and deployment scalable and straightforward. With the distinctive features of these two networking trends (VANET and SDN), the researchers move towards the SDN-based VANET system known as SDVN. For the better performance of SDVN, we believe that data security is a significant, challenging concern. Several security issues and threat vectors in SDVN may be victims of attacks on vulnerabilities, as shown in Figure 1. There may be a possibility of a man-in-the-middle attack in the first threat vector, and the second threat vector may be a possibility of existing forged or bogus traffic flows in the data plane. The third vector may be a victim of attacks on vulnerabilities in Road Side Units (RSUs). The third vector allows the attacker to cause disorder in the network by the weakness of forwarding devices. The most critical, due to which the network operation can be compromised, are threat vectors four and five, respectively. The attacker can easily control the network due to attacks on the control plane communication and SDN controllers due to attacks on controllers and some controllers’ vulnerabilities. The last threat vector can cause the requirement of trusted resources for forensics and remediation, which can agree for investigations and exclude quick and secure recovery modes for carrying the network back into a safe operating condition.

### 1.1. Contributions

The main contributions of this paper are as follows.

1. We highlight the network vulnerabilities and address the identified threat vectors to design a secure hierarchical architecture for SDVN with minimal resource utilization.
2. Our proposed novel and secure hierarchic architecture have improved the secure communication from vehicle to vehicle, vehicles to RSU, and vehicle to infrastructure. Moreover, we use the PKI-based digital signature scheme to secure communication between V2V and V2I to protect networks from adversaries attacks.
3. Additionally, we have used the concept of a three-way handshake mechanism to establish a reliable connection between the main and sub SDN controllers for a secure key generation along with forwarding secure data dissemination.
4. The proposed model is validated and proved using the simulation tool AVISPA for better performance.
5. Moreover, we have validated our proposed architecture’s fundamental security properties using a formal security method.

### 1.2. Paper Organization

The paper is organized as follows. Section 2 consists of related work about VANETs, covering the basic overview and its architecture, the overview of SDVN, security schemes proposed in SDVN, and VANETs. Moreover, we identify and highlight the possible issues and threat vectors. Section 3 consists of the proposed scheme. This proposed scheme consists of a network model and a proposed security mechanism for SDVN architecture that contributes to a novel, secure, and efficient hierarchical architecture. This section provides secure communication between V2V, V2 RSU, and V2I to protect networks from adversaries’ attacks. This section also provides deployment of the proposed efficient and secure SDVN architecture; Section 4 consists of the proposed secure scheme performance analysis. The proposed security analysis consists of two sections. The first section describes the formal proof of security model, and, in the second section, these security models are validated using AVISPA. We provide the comparative analysis in terms of cost analysis and the comparison of the security properties. Section 5 of the paper contributes to the conclusion and future work.

## 2. Related Work

In the following sections, we have presented a detailed literature review, such as SDVN and secure vehicular communication.

### 2.1. Overview of VANETs

VANET is an integral element of ITS and a particular MANET. There are three communication types of VANETs: V2V, V2R, and V2I. There are several communication technologies through which VANET can communicate, like DSRC that provide the opportunity for communication among ITS components (vehicles, infrastructure) and WAVE that defines MAC/PHY protocols and standards used for vehicular communication [1]. The leading working of VANET is roadside information processing through sensors, which is valuable for drivers to have safety while driving, accident avoidance, map location, weather forecast, etc. [1]. VANET consists set of nodes and a few RSUs. Other segments that are used in the VANETs are sensors, which consist of Onboard Units (OBUs) and Tamper Proof Devices (TPD) that help in sending the information to vehicles [4]. VANETs enable communication between V2V and V2R through the wireless medium called WAVE. Following are the components of VANETs and their communication, as shown in Figure 1.

**Node**: In VANET, nodes are vehicles that can communicate b/w other vehicles or roadside resources.

**OBU**: Through the OBUs, vehicles could communicate with each other directly or through RSUs deployed at different positions on the road.

**RSU**: RSUs are computing device that acts as gateways to access the Internet, located at different positions on the roadside, which provide connectivity support and link to a passing vehicle.

**TPD**: TPD provides secure storage space, speed, route, keys, and communication capability for the vehicle.

**TA**: Trusted authority has the responsibility to authenticate all the VANET users and manage their secret keys.

**Application Unit**: The applications may be located in the RSU or the OBU.

### 2.2. Overview of SDVN Architecture

Several SDVN architectures presented in the literature mainly focused on centralized SDVN architecture, partial decentralization having selected servers’ architecture, and hierarchic SDVN architecture. In this regard, M. O. Kalinin et al. in [2] presented the idea for designing SDVN architectures and their security. The authors focused on designing SDVN architectures and build three SDVN architecture, which mainly contributes to centralized SDVN architecture, Partial decentralization having selected servers’ architecture, and hierarchic SDVN architecture. Furthermore, for these architectures, the experimental evaluation is carried through the network simulator with NS-3, SUMO, and SDN Mininet-WiFi. SDVN is a new emerging technology that requires an efficient routing strategy. For this, Sadio et al. [5] proposed an SDN-based routing protocol on topology bases. Flow tables are created by SDN based on algorithms with the help of predicated topology. Two models are used for data communication which is unicast and geocast. From the simulation results, we concluded that SDN performance is better than that of traditional routing models. Soufian et al. [6] provided dynamic controllers in Software-Defined Vehicular Network. They developed a dynamic architecture for the placement of controllers for using traffic routines. There must be communication overhead using the network state to control and disseminate information from the central SDN controller. Due to this, they proposed a dynamic SDN controller. The proposed dynamic architecture is evaluated using real traffic and efficient compared to traditional routing protocols. Lionel et al. [7] presented Multi-access Edge Computing (MEC) for SDVN. The proposed model consists of two algorithms. Algorithm one consists of selecting the received information from nearby neighbors’ vehicle messages from V2V and V2I. The second algorithm is used for updating flow tables for forwarding devices. The architecture consists of four layers that reduced latency computation using routing paths. As we know, the SDVN is a new emerging technology; Khadir et al. [8] proposed a new offloading mechanism for SDVN integrated with fog computing to improve the efficiency and reduce the delay. Vehicles send a request for different services to fog devices on an on-demand basis. The fog devices give response to a vehicle within minimum time. However, in the meantime, some challenges such as offloading the mechanism to nearby fog nodes. However, this nearest fog node is busy sometimes, and the second one is the selection decision of the best Off Load Destination (OLD), where the fog node suffers from additional burden by acquiring the neighboring fog node information. To cope with this issue, a new offloading mechanism for SDVN integrated with fog computing is proposed and, for optimal selection of fog nodes, an SDN technology is used. The simulation result shows that the proposed scheme is efficient by taking less response time and significantly outpacing other offloading policies. In [9], Baihong et al. proposed SDN Based Vehicle Ad Hoc On-Demand, Routing Protocol (SVAO). They compare SVAO with other ad hoc routing protocols such as Optimize Link State Routing (OLSR), Dynamic Source Routing (DSR), Destination Sequence Distance Vector (DSDV), and Distance Based (DB) routing protocol through simulation. Based on the packet reception and packet delay analysis, the SVAO performs better than the others in large-scale networks or high vehicle speeds. In [10], Balamurugan proposed a scheme for VANETs using SDN technology in which the deployment of SDN in VANETs and its importance are discussed.

### 2.3. Security Schemes in SDN and SDVN

Different security architectures are proposed for solving security issues in SDVN. In this context Harsha et al. [11] proposed a framework to secure the communication in software-defined VANETs by providing an identification mechanism for malicious vehicles in a dynamic environment using a trust-based concept. For the detection of malicious vehicles, they used two algorithms for providing double security checks. The first algorithm is used to identify a trusted vehicle, and the second algorithm is used to identify malicious vehicles. The system shows better results in terms of improving the throughput and reduces the delay. Maxim Kalinin et al. [12] suggested a Software-Defined Security (SDS) approach for VANETs based on SDN technology. Overall, security is controlled, managed, and implemented by software throughout the entire network. SDN dynamically controls and manages network segmentation, intrusion detection, and access control using a programmable structure. Here are four functional layers for SDS implementation: security software, security policy management and orchestration, data layers, and virtualization. For SDS implementation, the author tried to achieve the best security, access control, and confidentiality in VANETs. Huijun Peng et al. [13] presented a method that finds the anomaly flows based on SDN to secure the SDN flows. The author gives an overview that provides the structure and the basic process flow to detect SDN anomalies. This method classifies an optimization for anomaly detection with a proposed algorithm that improves the detection and accuracy rate of detecting anomalies and reduces the false positive rate in an SDN environment. S. M. Mousavi et al. [14] proposed entropy-based quick Distributed Denial of Service (DDoS) detection against SDN controllers. In this scheme, the controllers are protected by allowing the controllers’ capabilities and calculating the entropy to receive grouping requests by controllers, which leads to the quick detection of identification anomalies, in [15] proposed an authentication scheme by introducing key insulation in VANETs to address security issues in different attacks on VANETs. Before signing the vehicle, it obtains its updated secret key with the help of TPD. First, the timestamp is checked whether it is valid or not, and then it matches the signature, either correct or not. With this, vehicles gain forward and backward secrecy also updates their secret keys periodically. VANET and SDN are the two most popular and necessary models for vehicular communications, as reflected from the literature review. It has some limitations which need to address and provide a possible solution for the efficiency of overall V2V and V2I communications. For better and secure communication, a security model is necessary. We believe that security is a significant, challenging concern for moving towards the design of an efficient and secure SDVN architecture.

In the scheme, ref. [16] authors applied the AVISPA tool concept to validate the proposed model’s security properties in an efficient and desirable way. Moreover, in scheme [17] authors designed a novel and efficient scheme based on proxy signcryption to authenticate the users and improve the security of the data during transmission on public networks. In the scheme, ref. [18] authors used ECC-based blind signcryption for mutual authentication and improved the security and privacy of the proposed architecture. In the scheme, ref. [19] used online/offline signcryption to reduce the cost overhead in terms of processing, communication, and storage in the resources constrained environment. Additionally, for data authentication, they applied the concept of modified signcryption efficiently.

### 2.4. Issues and Vulnerabilities in SDVN

**Figure 2:**
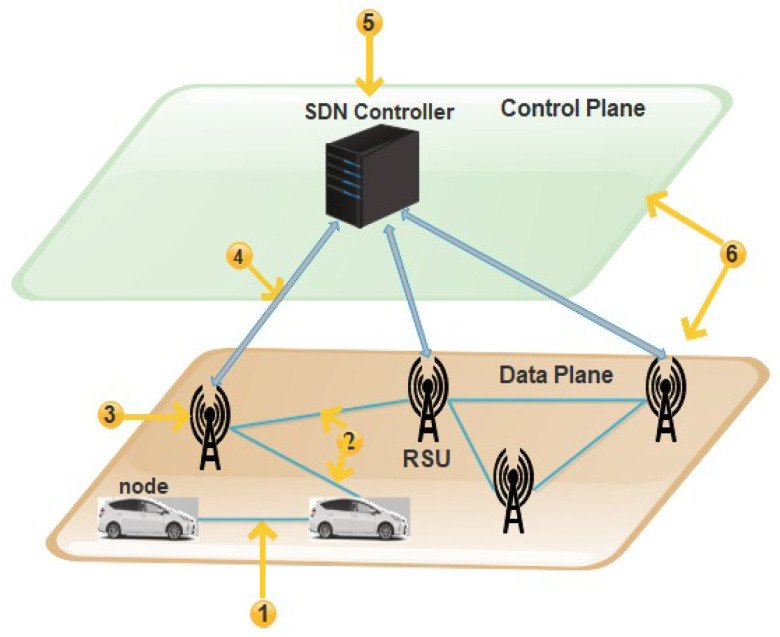
Issues and Vulnerabilities in SDVN.

SDVN environment is at risk due to several threats and vulnerabilities. These vulnerabilities are divided into six threat vectors shown in Figure 2.

These threat vectors found in SDVN may be a victim of attacks. In the first threat vector, there can be a possibility of a man-in-the-middle attack.

The second threat vector may suffer from fake or invalid traffic flows in the data plane. The nodes can be injected with fake information communicated to forwarding devices [20].

**Figure 3:**
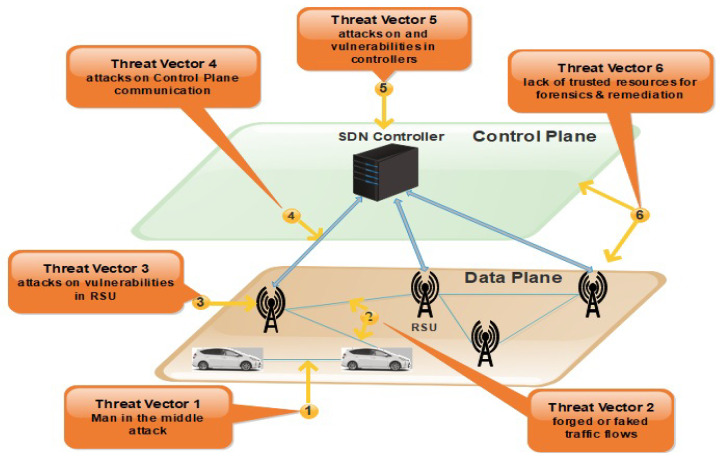
Issues and Threats Vectors in SDVN.

The detailed view of SDVN issues and threats vectors is shown in Figure 3. The third vector may be a victim of attacks on vulnerabilities in RSUs. This weakness of the forwarding devices may allow the attacker to cause disorder in the network. The Denial of Service (DoS) attack is faced by the forwarding plane in the SDN system due to the repetitive requests in VANETs nodes. Nodes are vehicles that have limited storage capacity. When packets coming to nodes and nodes does not find the path for that packet, a query is sent to the RSU to ask the controller about the missing rule. When the node receives the rule, they take a decision consequently. There may be an opportunity for a DoS attack in which a large amount of data is sent from the attacker’s side [21]. Threat vectors four and five are the most critical ones due to which the network operation can be compromised. The attacker can easily take control over the network due to attacks on the control plane communication. When multiple vehicles in the network send packets simultaneously to one another, a DDoS attack can be caused in the control plane because all the rules are not available on the switch. Therefore, multiple queries generated and sent to the controller causes a delay in the result of the dropping of queries [21].

The SDN controllers may be a victim of attacks due to vulnerabilities in controllers’ physical error. Another one is the generation of a fake controller. The malicious user can perform the original controller’s role known as identity spoofing, which sometimes forces the RSU to stop communication by dropping data [22]. In SDN, the entire network’s overall functionality will be affected when a single point of failure occurs in the controller while communicating with another device in a centralized system [21]. The last threat vector was identified between the control plane and the data plane but, in this paper, we address the security loopholes of threat vectors 1 to 5.

The depth studying of the above comprehensive literature identified several issues and threat vectors. We concluded to propose a secure communication mechanism for the SDVN environment that authenticates the entire network’s vehicle communications. To achieve and provide the essential security requirements and properties such as confidentiality, integrity, and non-repudiation to protect networks from adversaries’ attacks. Therefore, we move towards the design of efficient and secure software-defined vehicular network architecture to tackle this consideration.

## 3. Proposed Scheme

With VANETs and SDN’s rapid development as an emerging technology, SDVN has a tremendous attraction in academia and the research community. The vital feature of SDVN and its actual applications is still under consideration and development. Therefore, it is significant to design a secure architecture for SDVN to protect vehicles’ critical information from adversaries’ attacks during transmission on a public network. To cope with this issue, we propose an efficient and secure hierarchic architecture for SDVN. The network model and proposed security mechanism are discussed below. Table 1 shows symbols and descriptions used in this paper.

### 3.1. Network Model

**Figure 4:**
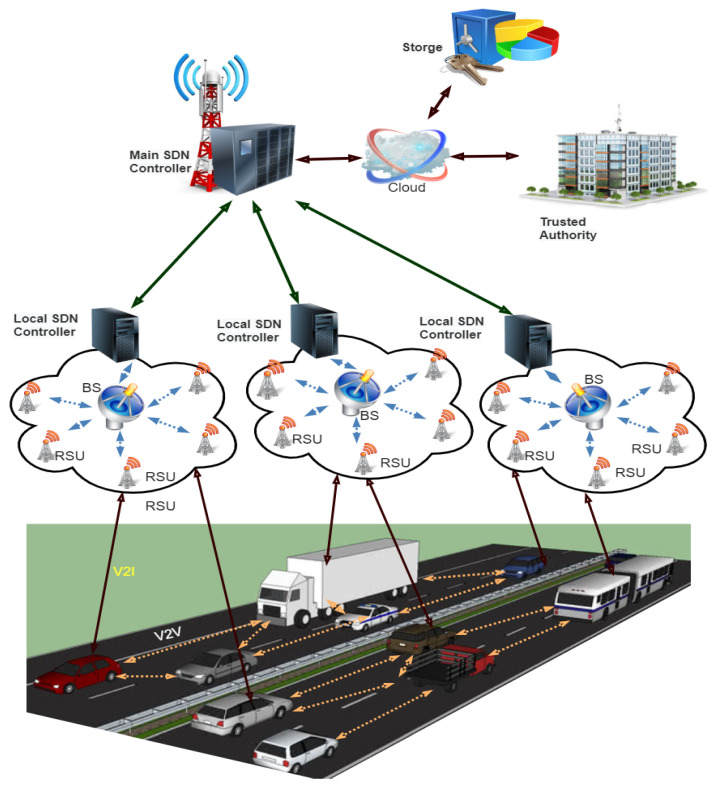
Hierarchic architecture for SDVN.

The proposed secure hierarchic architecture for SDVN is based on the work of [23]. This scheme proposes a secure hierarchic architecture for SDVN to secure traffic flow messages between V2V, communication of V2R, and between the SDN controllers as shown in Figure 4. The top-level of this architecture consists of the main SDN controller, and the lower level consists of the sub SDN controller, BS, and RSUs. The infrastructure layer consists of wireless switches and vehicles. A detailed description of hierarchic architecture for SDVN: the main SDN controller, sub SDN controller, RSUs, BSs, and vehicles are given below.

### 3.2. SDN Controller

The main SDN controller’s responsibility is to set the global rules to sub SDN controllers; the SDN controllers describe the network behavior, distribute the policy rules, and identify the routing parameters.

### 3.3. SDN Road Side Unit

It is a physical device that is placed along the roadside. It is a computing device that acts as gateways to access the Internet at a different position on the roadside, providing connectivity support and a link to a passing vehicle. The SDN controller monitors vehicles’ communication via periodical control messages and RSU status due to the global view network behavior.

### 3.4. SDN Nodes

In VANET, nodes are vehicles that can communicate between other vehicles or roadside resources.

### 3.5. Trusted Authority

The TA’s responsibility includes:

1 The registration of vehicles;
2 To authenticate the registered users to VANET and manage security parameters, including all the access parts.

### 3.6. SDN Cloud

This layer consists of the SDN cloud, where the SDN controllers are connected. Various computations are performed, such as the registration of vehicles and drivers, certificates generation for users, and keys exchange for users are performed, car speed calculations and distance, road traffic situation. All this information is stored and processed in a database and is managed through the cloud.

### 3.7. Proposed Security Mechanism

This section describes the proposed optimal architecture related to data security and privacy. We design a secure and efficient hierarchic architecture for SDVN with minimal resource utilization to protect vehicles’ critical information from adversaries’ attacks during transmission on a public network. Furthermore, in our proposed secure hierarchic architecture, we have improved the secure communication between vehicle to vehicle, vehicles to RSU, and infrastructure using PKI-based digital signature. Additionally, our proposed security mechanism consists of securing the communication between vehicles to vehicles, vehicles to RSU, and between sub SDN and main SDN controller. We have applied the concept of a three-way handshake mechanism to establish a reliable connection between the main SDN and sub SDN controller for a secure key generation and onward secure data dissemination. Moreover, the proposed architecture’s security properties were validated using formal security and AVISPA tools.

#### 3.7.1. Secure Communication between Vehicle to Vehicle

We use a PKI-based digital signature scheme to secure the communication between vehicles. An overview of PKI based digital signature is presented below;

#### 3.7.2. Secure Digital Signature

**Figure 5:**
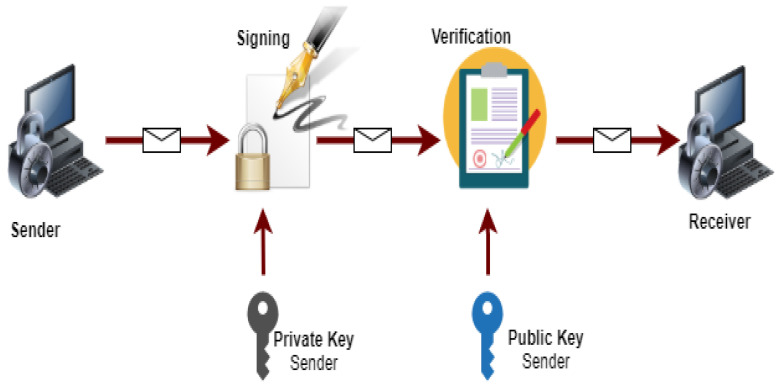
Working flow of digital signature.

The digital signature is a mathematical process of protecting the document from unauthorized users. It ensures that the digitally transferred data is authentic and validates that the document sent has no changes. The working flow of digital signature is shown in Figure 5.

Moreover, a digital certificate is signed and provided by the Certification Authority (CA) to guarantee trust in the signed data.

#### 3.7.3. Signing and Verification Process Using Proposed Digital Signature Algorithm

**Figure 6:**
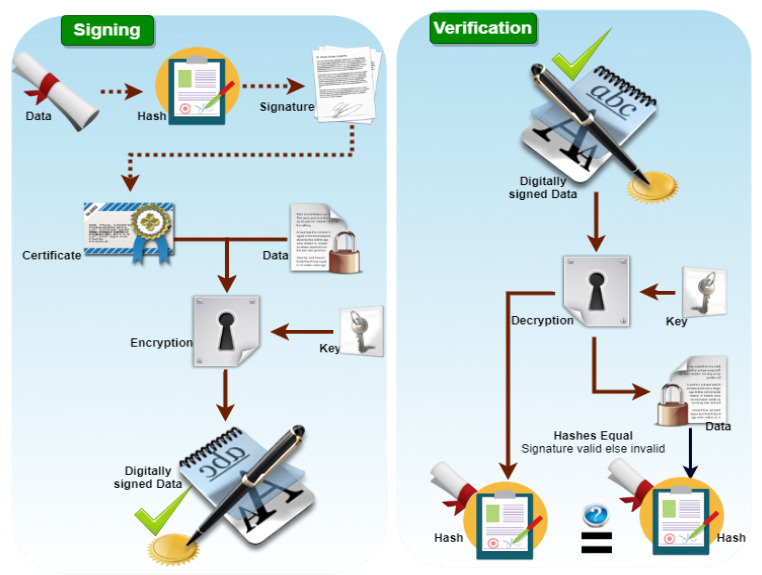
Signing and Verification process of Digital signature.

This section proposes a novel and efficient algorithm for signing and verifying the vehicle-sensitive information in the SDVN environment. Using this algorithm, we have achieved the elementary security properties of data authentication, integrity, and non-repudiation efficiently and desirably. Moreover, the master secret key of RSU and SDN controllers is established and distributed securely for further confidential communication among local SDN, main SDN, RSU, and vehicles. A nonce-based mutual authentication is performed between the data plan and control plan during data transmission from RSU to the main SDN controller. Furthermore, we have computed hash value using a hash function and other parameters to validate the vehicle information after transmitting to RSU. The vehicle’s computed private key is applied for signing, while the public key of the corresponding vehicle will be applied for verification on the destination side. The main SDN controller checks the data integrity of vehicle received data and computes a hash value. Compared with the received hash value, and if these hash values do not match, the received data is changed and does not remain in its original form; otherwise, the data is original and stored in the cloud for further decision-making of the SDVN environment. The signing and verification process is shown in Figure 6.

In a digital signature, we achieved the authentication and integrity of sensitive data. Initially, we have defined global public key components for the generation of user private keys. We select a random number (*x*) in the user private key that belongs to (*q*). Moreover, we calculate the user public key, where (*g*) is a generator and (*x*) is the selected random number that belongs to mod (*p*). We kept secret the security number (*t*) preserved the data’s privacy while using a signature algorithm to verify sender data on the receiver side. Furthermore, in the verification step, we authenticate the received data’s identity and the claimed sender. The below Algorithm 1 explained the proposed digital signature process in detail.
**Algorithm 1:** Proposed digital signature algorithm.  **Input:**
*n*,$V_{ID}$, ${RSU}_{ID}$  **Output:** Vehicles key pairs, i.e., public and private, the master secret key of RSU (${RSU}_{msk}$)  1.Global public key component $p:\mathrm{prime}\;\mathrm{number}\;2^{L-1}<p<2^{L}$  2.Deployed public parameters g,p,a and *b*  3.Choose random number x∈(1,2,3⋯q−1)  4.Computes vehicle private key $V_{Prk}=(x\cdot g)$  5.Again choose random number y∈(1,2,3⋯q−1)  6.Computes vehicle public key $V_{Puk}=(y\cdot g)$  7.Computes SDN controller master secret key ${SDN}_{msk}=k\cdot g$ mod *p*  8.Where *k*: any integer number (0<k<q)  Signature:  **For** all registered vehicles and RSU **do**  1.Assign unique ${ID}_{i}\;$ to each registered vehicles such as ${\;V}_{ID}$, RSU as ${RSU}_{ID}$  2.Computes pseudo ID of vehicle as H ($V_{ID}$ || K|| nj), j=1,2,3⋯n  3.Computes $C:y^{2}+h\left(x\right)y=f\left(x\right)$  4.Computes keys pairs of RSU such as ${RSU}_{prk}=(x\cdot g⨁\mathrm{Nonce})$  5.Computes $r_{sx}=r_{{sx}_{i-1}}⨁H\left(V_{r_{i-1}}\right)$  6.Applied the master key of SDN controller for signature  7.Computes public key of SDN controller such as ${SDN}_{puk}=\left({SDN}_{msk}\cdot g⨁\mathrm{Nonce}\right)$  8.Computes R=K·gmodq  9.Computes $S=[K^{-1}\left(H\left(M\right)+x\cdot R\right)]\;\mathrm{mod}\;q$  **End for**  Verifying:  1.$V=[\left(g^{u1}\cdot y^{u2}\right)mod\;p]\;\mathrm{mod}\;q$  2.$U_{1}=\left[H\left(M^{\prime }\right)\omega \right]\;\mathrm{mod}\;q$  3.$S=K^{-1}(H\left(M\right)+x\cdot R)$  4.R=K·gmodq  5.$\omega ={\left(S^{\prime }\right)}^{-1}$  6.$U2=[\left(R^{\prime }\right)\omega ]\;\mathrm{mod}\;q$  7.$V=R^{\prime }$

#### 3.7.4. PKI Based Digital Signature Scheme

**Figure 7:**
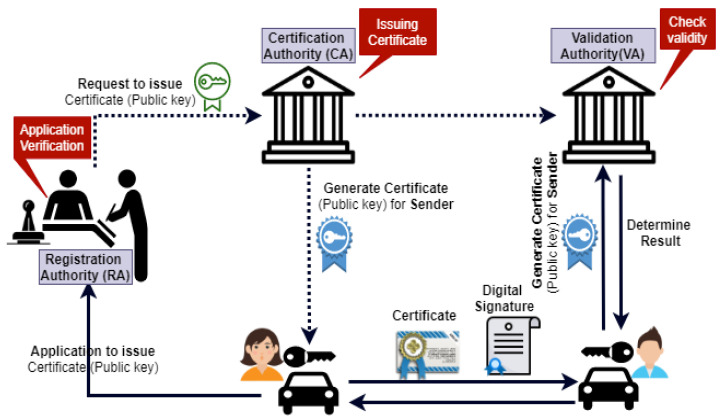
PKI based digital signature scheme for secure V2V communication.

The following steps are required whenever a vehicle wants to communicate with another vehicle, as shown in Figure 7.

1. The sender sends a request to the Registration Authority (RA) with their public key for issuing the certificate.
2. The RA verifies the sender’s request and forwards it to the CA.
3. The CA issues the certificate with their public key, stores this certificate to the repository, and sends a copy to Validation Authority (VA).
4. Then, this certificate is back sent to the sender.
5. After that, the sender sends this certificate along with a digital signature to the receiver.
6. When a recipient receives this certificate, it is further sent to the VA to check the certificate’s validity. The VA checks three things; first, it checks that the certificate is valid; if the certificate is valid, then it sends a message to a receiver that the certificate is valid; second, in case of the invalid certificate, the receiver will not regard the message; third, if the sender has no certificate validity at all, the receiver considers that this is the malicious user.
7. After checking the validity, the VA sends it back to the receiver.
  After the above process, secure communication will be established from V2V.

#### 3.7.5. Secure Communication between Vehicles and RSU

**Figure 8:**
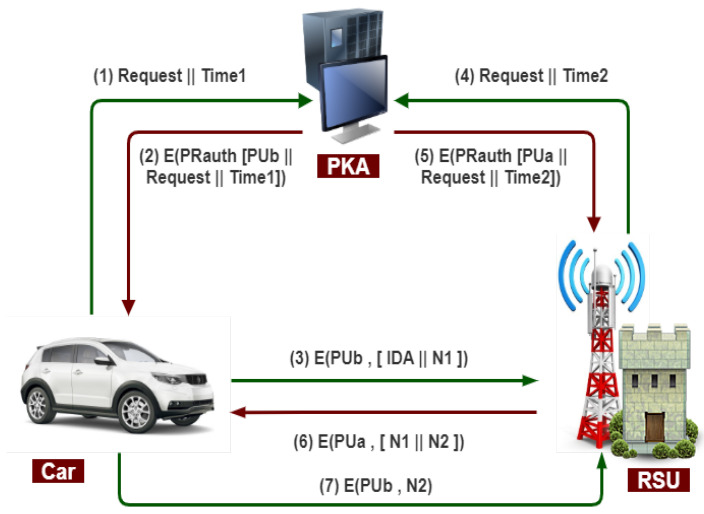
Public key authority infrastructure for secure communication b/w vehicle and RSU.

The public key authority provides the essential security for public key distribution that maintains an active directory of the public key for all members. The following process occurs, as shown in Figure 8.

1. The vehicle sends a message to a public directory that contains a request and timestamp for the current public key of RSU.
2. The public key authority responds to an encrypted vehicle message with the authority’s private key (PR-auth). The decryption of the message is done using the public key of the authority by the vehicle.
3. The message includes the public key of RSU, the original request, and the original timestamp.
4. The vehicle stores the RSU public key. For encrypting the message, an identifier of the vehicle (IDA) and a nonce (N1) are used for unique identification.
5. The RSU sends a message to a public directory containing a request and timestamp for its current public key.
6. As usual, the public key authority responds to the RSU message and retrieves the vehicle’s public key. In this way, the public keys have been securely delivered to the vehicle and RSU to protect an intruder’s communication.
7. When the RSU is sending a message to the vehicle using the public key of the vehicle (PUa) with a nonce (N1) and RSU generates a new nonce (N2) to assure that this vehicle and RSU are correspondents to each other.
8. With the help of the public key of RSU, the vehicle encrypts the message and returns nonce (N2) to RSU to ensure the exact correspondent.

Therefore, in this case, seven messages are required for secure communication between the vehicle and RSU.

#### 3.7.6. Secure Communication between Main SDN Controller and Sub SDN Controller

**Figure 9:**
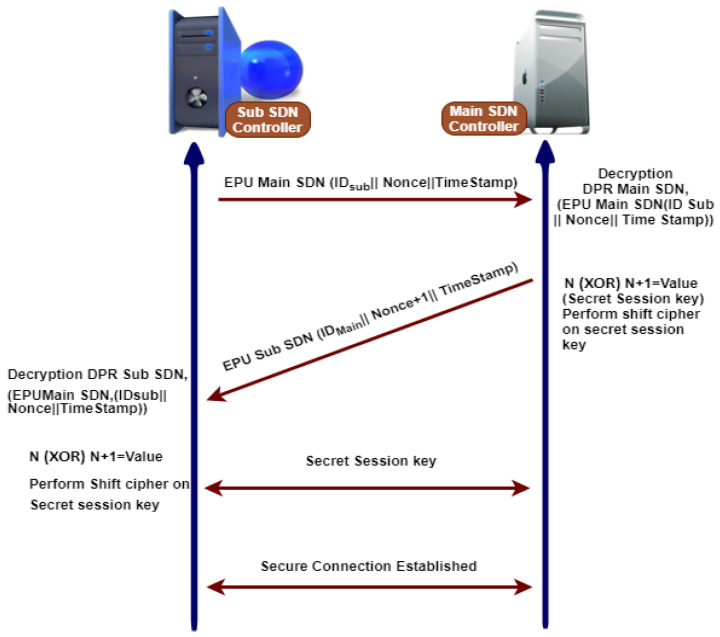
Three way hands shake mechanism for secure communication between sub and main SDN controller.

Whenever controllers are required to communicate with each other, the following steps are needed before starting the secure communication, as shown in Figure 9.

1. Any controller has its master keys like a master public key ($M_{PUK}$) and master private key ($M_{PRK}$).
2. Master public keys of both are exchanged publically.
3. The sub SDN controller sends a message to the main SDN controller that contains $ID_{\mathrm{Sub}}$, a nonce (*N*), and a timestamp that is encrypted with the public key of the central SDN controller.
4. The central SDN controller decrypts the message with their private key, gaining the original message, and responding sub SDN controller message that includes $ID_{\mathrm{Main}}$, timestamp, and adds one nonce (N+1) and is encrypted using the public key of sub SDN controller.
5. The sub SDN controller decrypts the message using their private key to gain the original message that contains $ID_{\mathrm{Main}}$, timestamp, and nonce plus one (N+1).
6. Therefore, the main and sub SDN controllers have one nonce (*N*) and nonce plus one (N+1). They perform an XOR operation on nonce values to produce a secret session key after establishing a secure connection.

### 3.8. Deployment of Proposed Efficient and Secure SDVN Architecture

This section explains the practical scenario of the proposed efficient and secure SDVN architecture, and we assume that several vehicles want to communicate with each other by sending information directly or through RSUs. It is essential to securely send and transfer this information between V2V, V2 RSU, V2I, and the SDN controllers. If a vehicle wants to communicate with other vehicles or through RSUs, the communication devices include the OBU, RSU, SDN controllers, and TA. OBU is used for vehicles’ direct communication, or they can use RSUs as a middle party. TA is providing security management, the security model of the proposed consists of the following three parts. (1) Vehicle registration (2) Vehicle authentication (3) Vehicle key update

The description of each phase is discussed below.

### 3.9. Registration Phase of Vehicle

In this phase, all the participating vehicles of SDVN are pre-registered with RSU. Moreover, the System administrator can efficiently and securely store the public keys and vehicle ID ($V_{ID}$) of all the registered vehicles on RSU and the public key, RSU ID ($R_{ID}$) of the nearest RSU stored on each vehicle.

### 3.10. Vehicle Authentication Phase

In our proposed efficient and secure SDVN architecture, the control plane establishes the authentication and routing policies to secure data transmission from the vehicle to RSU and vehicle to infrastructure. Furthermore, we have applied the PKI-based digital signature concept to authenticate the vehicle along with a pubic key for onward secure communication of information.

- In our proposed scheme for the authentication process, each vehicle sends its credential to the nearest RUS, such as vehicle ID ($V_{ID}$) and position area ($p_{i}$).
- The concerned RSU matched the pre-stored vehicle ID ($V_{ID}$) with received vehicle ID ($V_{ID}$) if both vehicle ID ($V_{ID}$ ) is matched, then authentication is granted. Otherwise, vehicles are isolated from the networks to protect the data from adversaries’ attacks.
- RSU verify the revocation ID polynomial, in case vehicle ID ($V_{ID}$) revoked then $Z_{n}^{i}\left(x\right)$= 0, then verify the location area ($p_{i}$), if the location is within the RSU range so generated random number ($R_{i}$). Additionally, to protect the SDVN from replay attack, we apply the timestamp ($T_{si})concept$ in our proposed architecture.
- When RSU received the encrypted message from the vehicle in a particular range, it will check the timestamp ($T_{si})$ validity within the allowable range; if timestamp $\left(T_{si}\right)$ is found to be correct, then connection established discard the connection.
- Vehicles generate the token (λ1) = ($V_{ID}\left|\right|p_{i}$), (λ2) = h$\left(R_{i}\left|\right|p_{i}||(T_{si})\right)$, and transmits toward the RSU. Then, RSU authenticates the token (ω=(λ1||λ2)) and random number ($R_{i}$).

### 3.11. Key Update Phase

In our proposed efficient and secure hierarchic architecture for SDVN, we have updated the session key after each round to maintain forward and backward secrecy of the networks and protect vehicle data from intruders’ attacks during transmission on public networks.

RSU performs the key updating phase, and CA provides a certificate for the authenticity of the public key used to encrypt vehicle messages. RSU concatenated the last round session key $\left(s_{n-1}\right)$ with last round message $\left(L_{m-1}\right)$ after hash function to computes new and updated session key, i.e., {$S=\left(s_{n-1}\right)⊕h(L_{m-1})$}.

The registered vehicles of the SDVN also updates the key by computing $d_{n-1}$ =$\frac{\left(s_{n-1}\right).\left(t_{n-1}\right)}{K(h\left(L_{m-1}\right))}$, where $\left(t_{n}\in V_{ID}\right)$.

## 4. Proposed Scheme Performance Analysis

This section presents the proposed security module formal proof and validates its security mechanism using a familiar simulation tool AVISPA.

### 4.1. Security Analysis

The security analysis section consists of formal proof of security model that validates the proposed architecture’s fundamental security properties using a formal security method. Moreover, the proposed security models are validated using AVISPA to proved the security of the proposed efficient and secure architecture.

#### 4.1.1. Formal Proof of Security Module

**Theorem** **1.** *Using theorem (1), we proved our proposed scheme’s confidentiality against adversary attacks, i.e., IND-CCA2.*

**Proof.**  We used the probabilistic polynomial algorithm against (IND-CCA2) in the random oracle model to satisfy our proposed scheme’s confidentiality. Using the DDHP assumption, we showed how Challenger (C) attacks a secure channel to tamper with the sensitive information transfer from the vehicle to RSU.

**Initial**:- Challenger (C) runs the setup algorithm using PKI-based digital signature to get the system parameters and compute the secure key for decryption.

1. $R=g^{k}\;\mathrm{mod}\;q$
2. S = $[K^{-1}\left(H\left(M\right)+\left(x.r\right)\right)]\;\mathrm{mod}\;q$
  Where k0<k<q
3. X=Randomnumberwhere0<x<q

**Phase.1:** Challenger (C) keeps secret the key ‘k’ and assume the key parameters to find the prime divisor of (P−1)

where $g=h^{(p-1)/q}\;\mathrm{mod}\;p$

where *h* is any integer lies 1<h<P−1

Now public key of the vehicle and other system parameters are transferred to the RSU by secure communication using the secret key.

**Attacker:** Initially, the attacker (A) performs the DDHP queries to get the random users *x*.

Where 0<x<q

If an attacker gets a valid random number, it will compute the private key; otherwise, the attacker cannot temper the secure communication between the vehicle and RSU. It is a computationally challenging problem for adversaries to get the valid random number *x*.

**Phase.2:** Attacker (A) used the queries of phase 1 as input and computed the session key using DDHP assumptions.

$S=\sum_{i=1}^{n}n\;⨁n+1$

Now perform shift cipher on compute session key (*S*). Furthermore, we define events, i.e., $e_{1},\;e_{2},\;e_{3},\;e_{4}$.

$e_{1}:\;\mathrm{Attacker}$ does not execute the session key query using random number *x*.

$e_{2}:Challenger\left(C\right)$ does not abort the PKI based digital signature queries.

$e_{3}:\;\;\mathrm{Attacker}$ (A) Choose the RSU identity during the challenge phase.

$e_{4}:\;Attacker\left(A\right)$ can guess the $PU_{a}\;$ and $\;PU_{a}\;$ using system parameter from public key authority.

Now Session key $\left(S\right)={\left(1-T\right)}^{qk}$, $S[e_{2}\left|\right|e_{1}{\;]=\left(1-T\right)}^{qk}$,

$S[e_{3}||e_{1}||e_{2}\;]$ ≥T, and $S[e_{4}||e_{1}||e_{2}||e_{3}\;]$ ≥ε

So $S[e_{1}\;\Lambda \;e_{2}\;\Lambda \;e_{3}\;\Lambda \;e_{4}\;]$ ≥$\;T\;{\left(1-T\right)}^{qk+qu}\varepsilon$

Now solving DDHP instance T≤t+O$\left(q_{u}\right)T_{n}+O\left(2q_{H1}+2q_{k}\right)tm$

□

**Theorem** **2.** *In our proposed scheme using theorem (2), we proved Unforgeability, i.e., (EUF-CMA).*

**Proof.**  We used a polynomial-time probabilistic algorithm against (EUF-CMA) in the random oracle model to satisfy our proposed scheme’s unforgeability property.

Using CDHP assumptions, we proved that Forger (F) used the non-negligible feature ε to forge the PKI-based digital signature between vehicle and RSU for secure distribution of public key certificate.

$\varepsilon^{{}^{\prime }}$ $\leq \varepsilon T{\left(1-T\right)}^{qk+n-1}$

$T\leq t+O\left(2q_{h1}+q_{k}+3q_{s}+n+1\right)T_{m}\;+O\left(q_{s}\right)tp$

Where $h_{i}\left(i=1,2,3,\ldots n+1\right)$

**Initial:-** Challenger (C) run the setup algorithm using PKI-based digital signature in time (T).

Challenger (C) applies the CDHP (P,aP,bP) queries to proved unforgeability.

**Phase.1:** Challenger (C) keeps the private key of the signer to protect the vehicle’s data using the digital signature algorithm.

Challenger (C) performs the setup algorithm and other system parameters.

$PU_{a}=g^{x}\;\mathrm{mode}\;P$

Where *x* is a random number chosen by vehicle during the key generation process

K= integer number

Where 0<k<q

1. $r=(g^{k}\;\mathrm{mod}\;p)\;\mathrm{mod}\;q$
2. $s=[k^{-1}\left(h\left(M\right)+x.r\right)]\;mod\;q$
3. $v=[y^{u2}\;\mathrm{mod}\;p]\;\mathrm{mod}\;q$
4. $u1=[h\left(M^{{}^{\prime }}\right)w]\;\mathrm{mod}\;q$
5. $w={\left(s^{{}^{\prime }}\right)}^{-1}\;\mathrm{mod}\;q$
6. $u2=[\left(r^{{}^{\prime }}\right)w]\;\mathrm{mod}\;q$
7. $V==r^{{}^{\prime }}$

Attacker (A) randomly select $x\;\varepsilon \;Z^{*}p$ and compute $Pr_{a}=\;g^{-x}d\;\mathrm{mod}\;P$ and returns session key $\left(S\right)={\left(1-T\right)}^{qk}$

Forgery (F) used the CDHP assumptions to execute the private key for the tempering of the digitally signed document of the vehicle.

If $x^{{}^{\prime }}=x$ accepted otherwise rejected (⊥)

For all 1≤i≤m, and C wants to get the system tuples $\left\{x,\;PU_{a},\;PU_{b},Pr_{a}\right\}$ from list and generates the following equations.

$e\left(h_{1},PU_{a},S\right)=e\left(S^{*},P\right)\;e(\sum_{i=1}^{n}\;h^{*}i,PU_{a}-P_{Pub}\;)$

$e\left(h_{1}^{*},Pr_{a},\;S\right)=e\left(S^{*},P\right)\;e(\sum_{i=1}^{n}\;h^{*}i,\;x^{*}i,\;PU_{a}-P_{Pub})$

Now Challenger (C) execute

$S={\left(\;\;h_{1}^{*}\right)}^{-1}(Pr_{a}-\;\sum_{i=1}^{n}\;h^{*}i,\;x^{*}i,\;PU_{b}\;)$

Furthermore, we will calculate the probability of (C) success using the following events.

$e_{1}:\;C$ does not execute the CDHP queries for session key generation.

$e_{2}:\;\left(F\right)$ execute a correct and non-trivial encoded text of vehicle.

$e_{3}:\;e_{2}$ happens, and $x_{i}=0<x<q$

If the above events happened, so (C) successful; otherwise fails.

Session key $\left(S\right)={\left(1-T\right)}^{qk}\;\geq {\left(1-T\right)}^{qk}$

$S[e_{3}||e_{1}\;]\geq \varepsilon$

$S[e_{3}\left|\right|e_{1}\;\Lambda \;e_{2}{\;]\;\geq T\left(1-T\right)}^{n-1}$

So that $S\left[e_{1}\;\Lambda \;e_{2}\;\Lambda \;e_{3}\;\right]\geq \;{\left(1-T\right)}^{qk}$

$\varepsilon T\;{\left(1-T\right)}^{n-1}=\varepsilon T\;{\left(1-T\right)}^{qk+n-1}$

Hence, we proved that our proposed scheme satisfied both the security properties of confidentiality and unforgeability using Theorems 1 and 2. □

#### 4.1.2. Security Models Validation Using AVISPA

**Figure 10:**
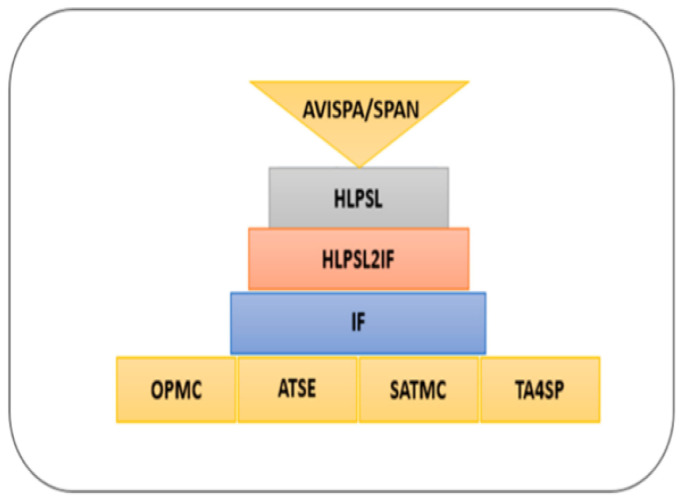
General Design Structure of AVISPA Tool [26].

The section contributes to secure the identified threat vectors and their vulnerabilities. We validate our proposed security scheme using a familiar simulation tool called AVISPA [24,25]. In AVISPA, the user can interact with a tool to identify the security problems to validate/verify and check the Internet’s sensitive security module and different cryptography techniques. It ensures that the proposed security model is SAFE/UNSAFE to code it into HLPSL. It is then converted to machine language by using the intermediate format. The prosed model has four modules, e.g., OFMC, CL-AtSe, TA4SP, and SAT-based module checker, to identify results. AVISPA simulation tool architecture is shown in Figure 10. The proposed SDVN architecture is secured using the PKI-based digital signature model for secure data transmission between V2V, public key authority infrastructure used for V2I, and a three-way handshake method to secure data transmission, SDN controllers.

**Figure 11:**
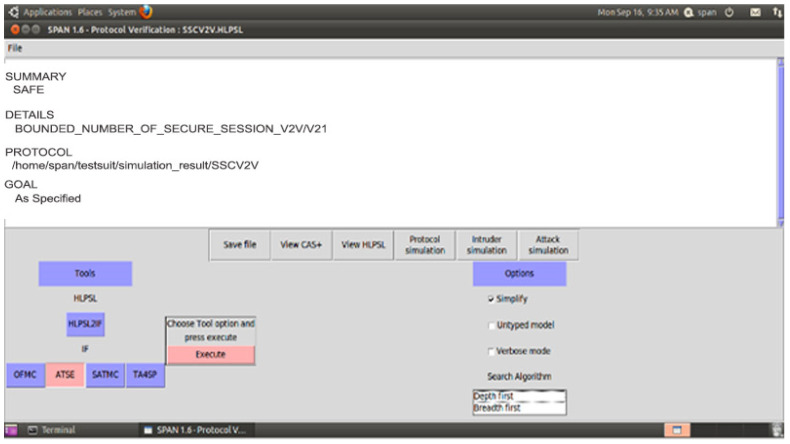
SSCV2V Simulation Result-1.

**Figure 12:**
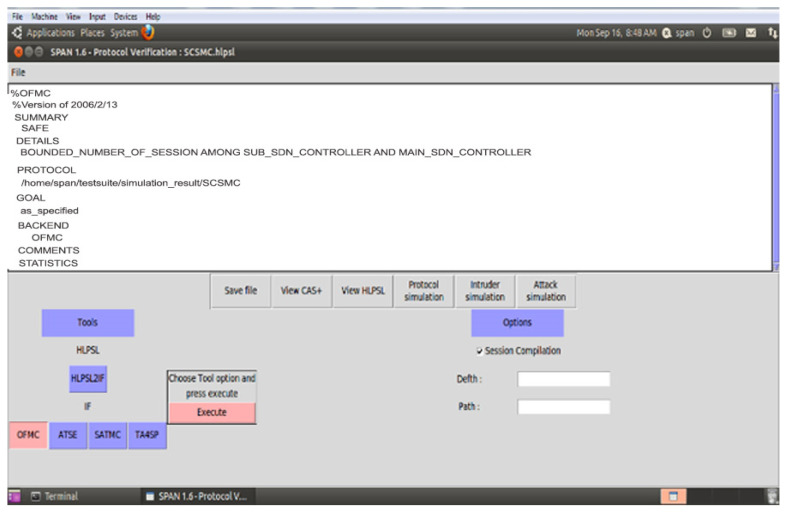
SCSMC Simulation Result-2.

The proposed security scheme Secure Session Communication between V2V (SSCV2V) is validated with AVISPA, and Figure 11 ensures that V2V and V2I are SAFE as well as achieve confidentiality, integrity, and non-repudiation property. For the secure communication between the sub and main SDN controllers (SCSMC) scheme, Figure 12 shows the simulation results SAFE.

### 4.2. Comparative Analysis

This section compares the proposed scheme security module with state-of-the-art schemes concerning security properties and costs in terms of transmission and processing cost.

#### 4.2.1. Security Properties Comparison

This section compares the proposed scheme security module with state-of-the-art schemes security properties reflect in Table 2.

1. **Mutual Authentication** (SP1): Authentication is the vital parameter for verifying participating vehicles for onward secure communication using public networks in the SDVN environment. In our proposed secure architecture, we have applied the PKI-based standard digital signature mechanism for mutual authentication among vehicle to vehicle, vehicle to RSU, and vehicle to infrastructure to isolate the networks’ illegal vehicles.
2. **Resist Intruder Attacks (SP2):** The use of a private key and other parameters such as random numbers and timestamps in the PKI-based digital signature process during authentication has protected the proposed SDVN architecture from intruder attacks.
3. **Provision of Anonymity (SP3):** We have achieved obscurity using valid and fresh tokens in the proposed secure architecture during the signing and verification process. Additionally, for secure communication among RUS and vehicles, new parameters such as random number, timestamp, location, and PKI-based, the private key will be generated to enhance the security and privacy of the proposed SDVN architecture.
4. **Protect Reply Attack (SP4):** To protect our proposed efficient and secure architecture from replay attack while disseminating information from vehicle to vehicle, vehicle to RSU, and vehicle to the infrastructure, we have concatenated the valid and fresh timestamp with data to protect reply attack. Furthermore, the participating nodes in the proposed SDVN can discard the late transmitted information to protect the targeted nodes from wrong decision-making during traveling.
5. **Protect Spoofing Attack (SP5):** In the proposed secure architecture of SDVN, the intruders cannot spoof the RSU, CA, and vehicles’ identity because the secure token is generated using the participating nodes’ private keys. Moreover, verification authority is used to verify the received public keys’ authenticity in the destination node.
6. **Data Authentication (SP6):** In the proposed PKI-based digital signature scheme for SDVN, we have authorized data source and integrity using assembled vehicle ID and key pairs to authenticate the data. If vehicle data is valid, it will be stored in the intelligent transportation system database for future decision-making activities; otherwise, it will be rejected. The concerned illegal vehicle is isolated from the VANET to improve the networks’ security and discard false transmission.
7. **Resist man in the middle Attack (SP7):** The concerned private and public vehicle credentials in the proposed PKI-based digital signature scheme are used to establish the authentication tokens. Moreover, after three-way handshaking, the secret session key is used to secure the transmission of information between vehicle to RSU and vehicle to infrastructure to protect the information from forge. At the same time, the intermediate nodes cannot access confidential information using an illegal way.
8. **Forward Secrecy (SP8):** To enhance the security of SDVN, in our proposed architecture, the computed secret session key is updated after a specific time interval, and adversaries cannot access the sensitive data of vehicles by guessing the previous session key of the networks.
9. **Backward Secrecy (SP9):** Using this feature in our proposed architecture, we have prevented the vehicle’s information from illegal usage. Furthermore, intruders cannot access the old transmitted information in case of session key exposure.

#### 4.2.2. Cost Analysis

**Figure 13:**
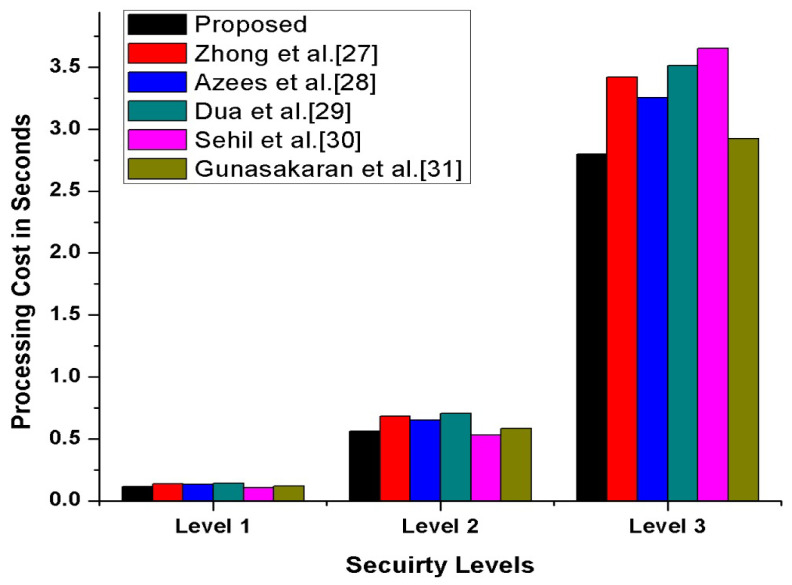
Comparative Computational Cost Analysis.

**Figure 14:**
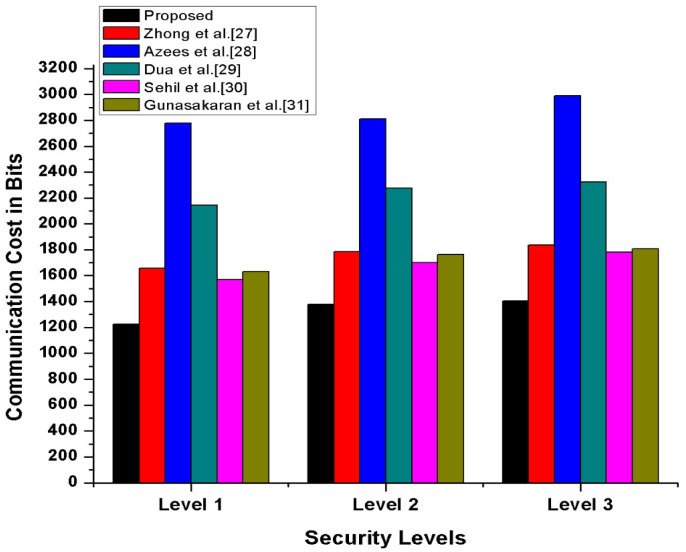
Comparative Transmission Cost Analysis.

In this section, we have compared our proposed scheme with other state-of-the-art schemes [27,28,29,30,31] in terms of transmission, processing cost, and the number of messages exchanges during transmission. Furthermore, to compute our proposed scheme’s operational cost, we have counted the major and costly operations. The costly operations are exponentiation, scalar multiplication, and bi-liner pairing, while the minor operations included hash function, addition, XoR, and subtraction, which is considered negligible. The experimental hardware specifications are ASUS Z-Book with an Intel R Core TM *i3*-6100 *U *CPU 2.1 GHz and 8 GB memory running on 64-bit Windows 8.1. According to [32], the per unit cost of scalar multiplication is 6.38 ms, and the per-unit cost of exponentiation is 11.20 ms while one bilinear pair is 20.01 ms. Therefore, we have calculated our proposed and other existing [27,28,29,30,31] schemes’ operational comparative cost analysis. The detailed comparative cost analysis is reflected in Table 3. According to Gunasakaran et al. [31], ECC multiplication, hash, symmetric encryption, and decryption are 0.0171 ms, 0.00032 ms, and 0.0056 ms. Therefore, as per these stated several unit operations, our proposed scheme, mutual authentication, consumes 0.112 seconds, which is also reflected in Figure 13. Additionally, we assumed that the vehicle identity =160 bits, timestamp =32 bits, location =32 bits and hash is 256 bits. Thus, as per this assumption, in our proposed scheme for authentication process, along with confidential transmission of vehicles, messages consumed (256+32)=288 bits, (512+32)=544 bits, (42+320+32)=394 bits, respectively, also shown in Figure 14. Therefore, in messages, transmission consumed a total of 1226 bits cost. Moreover, in our proposed scheme, we have maintained the tradeoff between security and cost to protect the SDVN information during transmission on public networks from adversaries’ attacks by utilizing minimal cost.

## 5. Conclusions

SDVN might be the future of VANETs to allow interoperability among diverse networks and efficiently manage the vehicle’s mobility. Moreover, according to the literature review, SDVN still suffered from security and privacy issues such as confidentiality, authentication, and access control. In this paper, security is the main research concern in designing secure SDVN architecture. We have focused on the vulnerabilities of the proposed SDVN architecture by addressing the identified threat vectors. In the proposed security model, we used a PKI-based digital signature model to secure data transmission from vehicle to vehicle, PKI for the vehicle to infrastructure, and a three-way handshake method for secure data communication in SDN controllers. Additionally, we have protected illegal SDN controllers from amending the performance, such as packet drops and redirection of deployed switches in the SDVN environment. Furthermore, we have demonstrated the benefits of our proposed architecture using the AVISPA simulation tool along with the formal security model; its efficiency is compared in terms of comparative analysis of security properties, cost analysis in terms of transmission cost, processing cost, and several messages which are exchanged during secure communication. In addition, we have ensured that our hierarchic architecture for SDVN is secure and satisfies all the basic security properties such as confidentiality, authentication, integrity, and non-repudiation in an efficient and desirable way.

In the future, it is possible to provide an appropriate mechanism for the last threat vector that can cause the requirement of trusted resources for forensics and remediation, which can agree for investigations and exclude quick and secure recovery modes for carrying the network back into a safe operating condition.

## Figures and Tables

**Table 1:** Symbols and Descriptions.

Symbol	Description
OBUs	Onboard Units
TPD	Tamper Proof Devices
MEC	Multi-access Edge Computing
OLSR	Optimize Link State Routing
DB	Distance Based
SDS	Software-Defined Security
IND -CCA2	Indistinguishable Chosen Ciphertext Attack
EUF-CMA	Existentially Unforgeable Chosen Message Attack
DDHP	Disional Deffie Helleman Problem
SSCV2V	Secure Session Communication between V2V
SCSMC	Secure communication between the sub and main SDN controllers
PR-auth	Authority’s private key
IDA	Identifier of the vehicle A
N	Nonce
PUa	Public key of the vehicle
*MPUK*	Master public key
*MPRK*	Master private key
*ID*Main	ID of Main SDN controller
*RID*	RSU ID
*VID*	Vehicle ID
*pi*	Position area
*Tsi*	Timestamp
*Ri*	Random number
*l*1	Token
*sn*-1	Last round session key
*Lm*-1	Last round message
*p*	Prime number
*x*	Random number
*VPrk*	Vehicle private key
*VPuk*	Vehicle public key
*SDNmsk*	SDN controller master secret key
*RSUprk*	RSU private key

**Table 2:** Comparative Analysis of Security Properties.

Scheme	SP1	SP2	SP3	SP4	SP5	SP6	SP7	SP8	SP9
Zhong et al. [27]	✕	✓	✓	✓	✕	✓	✓	✕	✕
Azees et al. [28]	✕	✓	✓	✕	✓	✕	✓	✓	✕
Dua et al. [29]	✓	✕	✓	✓	✕	✓	✓	✓	✕
Sehil et al. [30]	✓	✓	✕	✓	✓	✕	✕	✕	✕
Gunasakaran et al. [31]	✓	✕	✓	✕	✓	✓	✕	✓	✕
Proposed architecture	✓	✓	✓	✓	✓	✓	✓	✓	✓

**Table 3:** Comparative cost analysis.

Scheme	Transmission Cost (in Bits)	Processing Cost (in Seconds)	No. of Messages Exchanged
Zhong et al. [27]	823 ${}^{*}$ 832 n ${}^{**}$	0.0171 n + 0.1197 ${}^{**}$	1
Azees et al. [28]	7488	0.1302!,(n+1)0.0171+(n+4)0.0192!!	1
Dua et al. [29]	2144	0.1406	3
Sehil et al. [30]	1568	0.1061	5
Gunasakaran et al. [31]	1632	0.117	4
Proposed Architecture	1226	0.112	3

${}^{*}$ verification of single message. ${}^{**}$ batch messages verification. ! single signature along with a certificate. !!n no. of certificates and signatures.

## Data Availability

Not applicable.
